# Reduced patient restrictions following total hip arthroplasty: study protocol for a randomized controlled trial

**DOI:** 10.1186/s13063-015-0901-0

**Published:** 2015-08-18

**Authors:** Anil Peters, Miranda Tijink, Anne Veldhuijzen, Rianne Huis in ‘t Veld

**Affiliations:** Center for Orthopedic Surgery OCON, Geerdinksweg 141, 7555 DL Hengelo, The Netherlands

**Keywords:** Arthroplasty, Replacement, Hip, Posterolateral surgery, Precautions

## Abstract

**Background:**

Total hip arthroplasty (THA) is a very common procedure in orthopedic surgery. In the Netherlands, 25,642 primary THAs were performed in 2013. Postoperative hip dislocation is one of the major complications and has been reported in 0.5 to 10.6 % of patients after primary THA.

Several reports regarding the use of an anterolateral surgical approach have shown that a non-restriction or reduced restriction protocol does not increase the dislocation rate. For the posterolateral surgical approach it has been suggested that patient restrictions might be unnecessary but the amount of available literature is scarce. As such, randomized controlled trials aimed at investigating restrictions following THA using a posterior approach are strongly recommended.

The aim of this prospective randomized controlled trial is to investigate the non-inferiority hypothesis concerning the early dislocation rate after THA in patients with and without the use of a reduced restriction protocol.

**Methods/Design:**

After providing informed consent a group of 456 patients with symptomatic coxarthrosis will be randomized to receive a THA either with care as usual, i.e. receiving postoperative restrictions including the advice to sleep in a supine position for the first 8 weeks postoperatively, or reduced restrictions with no recommendations regarding the position during sleeping. Primary outcome measure will be the percentage of early dislocations within the first 8 weeks after THA. Secondary outcome measures will be patient satisfaction, time to functional recovery, quality of sleep and patient’s self-reported compliance with postoperative instructions.

**Discussion:**

To our knowledge this will be the first randomized controlled trial that compares a reduced restriction protocol with a restricted protocol following THA using a posterolateral surgical approach. Our hypothesis is that a reduced restriction protocol following THA with use of a posterolateral surgical approach has no influence on the early dislocation rate compared to a restricted protocol. Instead, embracing a reduced restriction protocol might even contribute to a higher quality of sleep, thereby facilitating a faster uptake and return to daily functions in patients after THA.

**Trial registration:**

ClinicalTrials.gov NCT02107248, registration date 3 April 2014.

## Background

Total hip arthoplasty (THA) is a very common procedure in orthopedic surgery [1]. In the Netherlands, 25,642 primary THA implants were performed in 2013 [2]. Postoperative hip dislocation is one of the major complications and has been reported in 0.5 to 10.6 % of patient after primary THA [3, 4]. Surgical technique and approach as well as implant selection, implant positioning, patient education and patient-related factors have an impact on the incidence of dislocations [5–7]. Traditionally, patient restrictions following THA are prescribed in order to prevent early dislocation by limiting the flexion of the hip (<90 degrees) as well as adduction and internal rotation [8]. In modern orthopedic surgery, less invasive, tissue-sparing techniques have been introduced and patients are operated upon using shorter acting anesthetics. Nowadays, surgery duration is shorter and patients are being mobilized early after surgery. These factors possibly contribute to less loss of muscle strength after surgery, resulting in a more stable hip joint immediately postoperative. Postoperative joint stability is further enhanced by the use of larger diameter femoral head components. Patients are also better educated and managed with clinical pathways which include detailed protocols, thereby reducing the risk of an early dislocation [6, 9]. However, the aspect of evidence-based application of restrictions after THA has attracted less attention, with long-standing protocols continuing to be routinely used in most hospitals.

Several studies have shown that no or reduced restriction protocols do not result in increased dislocation rates [10–12].

Moreover, Talbot et al. have documented that patients had difficulties sleeping and felt discouraged during the time of restricted postoperative hip precautions [12]. Likewise, faster return to normal activities, higher patient satisfaction and earlier return to work are the benefits that have been shown when using no or reduced restrictions following THA, making it a cost-effective and patient-friendly alternative to the restricted protocol [10, 13]. Nowadays, most of the available knowledge on the effects of reduced postoperative precautions and restrictions after THA has been obtained utilizing an anterior surgical approach [10–12, 14]. This might be explained by the fact that THA utilizing a posterior approach without repair of the posterior capsule and external rotators is associated with an increased risk for dislocation when compared to patients undergoing an anterior or anterolateral approach [15]. However, when conducting a posterior soft tissue repair this “increased” dislocation rate through the posterior approach is reduced [16]. As such, further research on applying a reduced restriction protocol in this group of patients is warranted [17].

To our knowledge this will be the first randomized controlled trial (RCT) that investigates use of a reduced restriction protocol following THA with use of a posterolateral surgical approach. Our hypothesis is that a reduced restriction protocol following THA with use of a posterolateral surgical approach has no influence on the early dislocation rate compared to a restricted protocol.

## Methods/Design

### Study design

The study design is a single***-***center, parallel-group, stratified, randomized trial with a planned duration of 3 years in which 456 primary THA patients will be allocated to either a care-as-usual group receiving postoperative restrictions including the advice to sleep in a supine position for the first 8 weeks postoperatively or an experimental group, receiving postoperative instructions without restrictions on sleeping position after THA surgery. The experimental group does not use a pillow between the legs in any sleeping position and the care-as-usual group, that is only allowed to sleep in a supine position, is advised to use a pillow between the legs while doing so.

A non-inferiority design was chosen as a standard approach to assess similarity of results of the experimental as opposed to the care-as-usual treatment. In order to avoid an imbalance in treatment assignments and to reduce the opportunity for bias and confounders, a stratified blocked randomization technique will be applied with random sequences of varying block sizes (varying from *n* = 2, *n* = 4 or *n* = 6). Among the stratification factors are operating surgeon and the preferred self-reported sleeping position (supine, prone, on the side, combination/no clear preference) of the patient. Measurements will be taken at baseline (preoperatively), 8 weeks (at regular polyclinic visit) 6, 12 and 24 months after surgery (postoperatively).

### Setting

Written informed consent, from patients who meet the inclusion criteria for participation in the trial, having verified that the candidate fully understands what is involved, will be obtained by the research nurse.

Patients will be recruited by the Center for Orthopedic Surgery OCON, Hengelo, The Netherlands. All THAs will be performed by one of OCON’s four orthopedic surgeons specialized in hip surgery and with at least 5 years’ experience in THA. Time, duration and type of anesthesia will be recorded in our patient database. The surgical approach is standard posterolateral with use of a capsular repair. The soft-tissue tension is optimized through neck-length adjustments until an axial force with the leg in extension produces 1 to 2 mm of soft-tissue laxity for male patients and 2 to 3 mm of soft-tissue laxity for female patients.

During trial reduction, stability of the hip is tested in full flexion; 90 degrees of flexion and 45 degrees of internal rotation; full extension; 0 degrees of extension and 45 degrees of external rotation; and with and without knee flexion (up to 90 degrees). Such stability testing is routine in our practice and was not modified for the present study. The implants that will be used are: Exceed ABT Ringloc-XShell, Biomet Orthopedics, E-Poly Hi-Wall Liner Biomet Orthopedics, Modular Taperloc complete femoral stem Biomet Orthopedics, Biolox Delta Modular Ceramic Head 32 mm (Dordrecht, The Netherlands). The postoperative protocol is full weight bearing to tolerance from day 1.

The first radiologic assessment, to control prosthetic positioning occurs on the day of the operation, with an anteroposterior (AP) and lateral view of the hip. The second radiographic assessment is at 8 weeks postoperative with an AP pelvic view and a lateral view of the hip. Implant position including cup inclination angle, acetabular component anteversion, hip offset, and leg length will be measured on the 8 weeks postoperative AP pelvic view. The target zones for anteversion and inclination are defined as 10–30 degrees and 30–50 degrees, respectively.

The local Medical Ethical Committee approves the study design, procedures, protocols and informed consent. The trial is registered at ClinicalTrials.gov NCT02107248.

### Study population

Patients with symptomatic osteoarthritis of the hip who are planned for THA are included if they meet the following criteria: ASA-classification I or II (American Society of Anesthesiologists); written informed consent provided by the patient.

Exclusion criteria are: blindness, scheduled second THA within 6 months, mental incapacity, or inability to fill in the questionnaires in Dutch, infection involvement, wheelchair-dependency, alcohol abuse, and neurological disorders such as Parkinson’s disease, or stroke and hypermobility syndromes such as Ehlers-Danlos syndrome.

### Interventions

After randomization, patients will receive either care-as-usual; postoperative instructions with the advice to sleep in a supine position for the first 8 weeks postoperatively or reduced restrictions; postoperative instructions without any restriction on sleeping position. All patients are instructed by the physiotherapist by oral and written guidelines which include the following:

- Not to cross the legs
- Not to squat
- Not to internally rotate the hip more than 45 degrees
- Not to flex the hip more than 90 degrees and
- Not to make a combination of these two movements
- When sitting not to use a very low chair that makes the hip flex more than 90 degrees
- When sleeping only to sleep in the supine position and to use a pillow between their legs during sleep
- When bending move the operated leg backward so the operated hip will not flex more than 90 degrees

### Care-as-usual/restricted group

In the restricted group patients have to sleep in a supine position during the first 8 weeks. Hip flexion over 90 degrees and internal or external hip rotation more than 45 is not allowed for the first 8 weeks. Patient will be mobilized by the physiotherapist at the day of the operation or the first postoperative day with full weight bearing to tolerance.

### Experimental/reduced restricted group

In the reduced restricted group patients are allowed to sleep in any position they find comfortable. Hip flexion over 90 degrees and internal or external hip rotation more than 45 degrees is not allowed for the first 8 weeks. Patient will be mobilized by the physiotherapist on the day of the operation or the first postoperative day. Patient will be mobilized by the physiotherapist at the day of the operation or the first postoperative day with full weight bearing to tolerance.

Patients are instructed by the physiotherapist how to lie in bed and how to prevent more than 90 degrees of hip flexion and 45 degrees of rotation. Next to the general instructions as stated earlier, patients in this experimental group receive additional instructions on how to lie and turn in bed by preventing more than 90 degrees of hip flexion and 45 degrees of rotation.

### Main study parameters/endpoints

Primary outcome measure is the difference in dislocation rate, expressed as a percentage, within the first 8 weeks between the group that receives postoperative instructions with the advice to sleep in a supine position for the first 8 weeks postoperatively and the group that receives postoperative instructions without any restriction on sleeping position. The diagnosis of a dislocated hip will be confirmed by clinical examination and X-ray findings.

### Secondary study parameters/endpoints

Secondary outcome measures are patient’s compliance with postoperative instructions, the influence of sleeping position restrictions on quality of sleep [18], the influence of sleeping position restrictions on patient satisfaction (anchor questions rating the degree of perceived quality of sleep and burden of the restrictions prescribed, the Client Satisfaction Questionnaire) and the effects of the advice to sleep in a supine position for the first 8 weeks on functional recovery.

### Other study parameters

These parameters include:

1. Resumption of specific activities following THA such as driving a car.
2. The use of assistive devices (e.g. pillow between the legs during sleep, crutches).
3. The self-reported compliance of patients with the restrictions prescribed.
4. Satisfaction with received quality of care (Client Satisfaction Questionnaire).

### Study procedures

After inclusion and prior to surgery, subject patients will complete the baseline questionnaire at the outpatient clinic during the intake by the nurse practitioner. The physiotherapist will mobilize patients on the day of the operation or the first postoperative day. During this mobilization patients are instructed how turn in bed and how to prevent more than 90 degrees of hip flexion and 45 degrees of rotation. The only difference in instruction between the two study arms is the sleeping position. When discharged to home or nursing home, patients receive a booklet describing the instructions relevant to the study arm they are assigned to. This booklet serves as an enchiridion for partners, physiotherapists, and other persons involved in the care of the patient. In addition, patients are handed a standardized diary booklet in which they are asked to document their sleeping position, exercise activities, experience of pain and any further comments they may want to convey. Patient will visit the outpatient clinic preoperatively and then at 8 postoperative weeks. Routine physical examination will be performed by the orthopedic surgeon. All scores and measurements will be recorded by the nurse practitioner who will also collect the diary booklet from the patients. Preoperative functional assessment will be done by pain severity (Visual Analogue Scale: range 0–10), hip function (Hip Disability and Osteoarthritis Outcome Score) and quality of life (EQ-5D). At 8 weeks follow-up the same functional assessment will be recorded by the nurse practitioner. This visit is combined with the regular postoperative control by the orthopedic surgeon. At 6, 12 and 24 months there will be follow-up questionnaires by Email, paper or telephone depending upon the availability of the patients’ Email addresses. The primary outcome: dislocations within the first 8 weeks, will be recorded in the patient’s file when visiting the emergency department.

### Sample size calculation

The maximum allowable difference in proportion in which there is still equality in the effect is not known in the literature. Previous studies suggested “a threefold difference in dislocation rate” to be a clinically relevant difference [4]. Since the literature is suggesting an average dislocation rate of 2.03 % [8] in the posterolateral surgical approach, a dislocation percentage between 2.03 and 6.09 % is considered to be “equal.” Hence, the planned sample size is *n* = 456 THA patients. (One-sided, *α* = 0.025, *β* = 0.80, missing data 20 %).

### Statistics

#### Populations

Primary analyses will be performed for both the intention-to-treat (ITT) and per-protocol (PP) populations. The PP population of patients will comprise those who completed all measurements and did not have any reasons for exclusion from this population, including no baseline data, no data at 8 weeks and/or 6 months or major protocol violations (e.g. position compliance < 80 %).

Additional analysis will be based on an ITT (All Patients Treated) population that consists of all randomized patients who had both a baseline and at least one post-baseline measurement.

#### Primary analysis

Descriptive statistics were calculated for all variables. Depending on the type of data were compared between the groups using either a *t* test (continuous) or chi-square (categorical).

The intervention group that receives postoperative instructions without any restriction on sleeping position will be declared non-inferior to the group that receives postoperative instructions with the advice to sleep in a supine position for the first 8 weeks postoperatively in case it can be demonstrated that the difference between these 2 groups does not exceed “a 3-fold difference in dislocation rate” in favor of the supine sleeping position group. This margin corresponds to the definition of clinically relevant difference in dislocation rate by Peak et al.

#### The secondary analysis

A repeated measure analysis of (co)variance (ANOVA; group and time) will be applied in order to investigate differences between the groups satisfaction, functional recovery (HOOS) and quality of life (EQ-5D) at 8 weeks, 6 months, 1 and 2 years postoperatively. In addition, correlation coefficients will be calculated between quality of sleep and functional recovery and quality of life. ANCOVA will be conducted dependent upon the analysis of differences in baseline characteristics between groups (e.g. preoperative values, age, radiographic analysis). Subgroup analysis will be directed towards identifying differences in functional recovery between patients with and without compliance and patients who were and were not satisfied.

#### Missing data

Missing values (<30 %) in the APT analysis will be handled by multiple imputation techniques. It will be assumed that any missing data will occur at random and missing values will be imputed for the ITT population using multiple imputation by chained equations. The imputation models will be specified to include the individual scores observed at 8 weeks, 6 months, 1 and 2 years and any available variable that has a statistical association with the outcome to be imputed or with “missingness,” as identified in a logistic regression analysis with “missingness” as the dependent variable. Corresponding to the percentage of missing values (with a minimum of 25) datasets with imputed plausible values will obtained, with 50–100 iterations between datasets. Predictive mean matching will be employed to obtain pooled parameter estimates and their associated standard errors for all analyses.

The results of the analysis of the primary study hypothesis using the pooled results will be compared to the results obtained on the observed (PP) data alone.

### Ethical considerations

This study is approved by the medical research ethics committee. The Medical Ethics Committee Twente acts as central ethics committee for this trial (Number P13-.31, NL4670604414). An insurance which is in accordance with the legal requirements in the Netherlands (Article 7 WMO and the Measure regarding Compulsory Insurance for Clinical Research in Humans of 23 June 2003) has been obtained. This insurance provides cover for damage to research subjects through injury or death caused by the study. Once a year, information will be provided to the medical research ethics committee on the numbers of subjects included and numbers of subjects that have completed the trial, serious adverse events/serious adverse reactions, and other problems.

## Discussion

Various postoperative restrictions have been proposed for patients undergoing THA to prevent early hip dislocation by emphasizing the importance of avoiding extremes of motion as well as to protect the soft tissue repair [8]. However, the scientific rationale for the effectiveness of these postoperative restrictions in the prevention of early dislocations is limited. Moreover, there are indications that applying a reduced restriction protocol has several benefits. In a prospective, randomized study, with use of an anterolateral surgical approach, Peak et al. reported that patients were much more satisfied when they were given fewer restrictions [10]. Furthermore, these patients achieved a faster return to daily functions and were able to return to work faster [9]. In addition, sleep was positively affected by the reduced precautions [10]. Ververeli et al. demonstrated that reduced hip precautions can facilitate recovery and are more cost-effective [14].

Today the available knowledge is mainly directed at analyzing the anterior or anterolateral surgical approach. However, there are two cohort studies that have investigated the effectiveness of a reduced restriction protocol in primary THA following a posterolateral instead of an anterolateral approach but these studies lack a randomization procedure. For example, Mikkelson et al. found no difference in dislocation rate comparing two cohorts with and without restrictive motion [13]. The study did not show any beneficial effect of rehabilitation without movement restrictions on patient evaluated function. Schmidt-Braekling et al. showed, in a retrospective analysis, that shortening standard posterior hip precautions from 6 to 4 weeks after primary THA utilizing a posterior approach does not increase the risk for postoperative dislocation within the first year after surgery [7].

In the present study we will compare two postoperative protocols that differ in the restriction of sleeping position following THA via the posterolateral surgical approach. Based on the study of Talbot et al., who found that patients had difficulties sleeping following a restricted protocol, our intervention group will not be restricted to sleep in a supine position [12]. In previous studies only the duration of the restrictions was shorted or the reduced restriction protocol was directed towards movement restrictions and use of assistive devices [7, 13].

Another interestingly aspect of the current study compared to the existing literature is that fact that it incorporates an analysis of the patient self-reported compliance to postoperative restrictions. Our study plans to assess patient compliance to the sleeping position instructions in relation to their preferred sleeping position.

In summary, the aim of this RCT is to show that a reduced restriction protocol following THA with use of a posterolateral surgical approach has no influence on the early dislocation rate. By omitting the postoperative sleeping restriction patients might have a better quality of sleep, higher patient satisfaction and a faster functional recovery. To our knowledge this will be the first RCT that investigates use of a reduced restriction protocol following THA with use of a posterolateral surgical approach.

## Trial status

Recruiting as of April 2014.

## Abbreviation

APT: All patients treated
